# Draft Genome Sequence of *Oleiagrimonas soli* 3.5X^T^, a Type Species in a Newly Identified Genus, Isolated from an Oil Field in China

**DOI:** 10.1128/genomeA.00469-15

**Published:** 2015-05-14

**Authors:** Yong Huang, Tingting Fang, Hui Wang, Haiyan Zhou

**Affiliations:** State Joint Laboratory on Environmental Simulation and Pollution Control, School of Environment, Tsinghua University, Beijing, People’s Republic of China

## Abstract

*Oleiagrimonas gudaosoli* 3.5X^T^ was isolated from an oil field and identified as a new member of a novel genus. The draft genome sequence of this strain, which comprises 3,379,958 bp encoding 3,010 open reading frames (ORFs), can provide insight into the life style of this newly identified genus in petroleum-contaminated soil.

## GENOME ANNOUNCEMENT

*Oleiagrimonas soli* 3.5X^T^ (=NBRC 110685^T^ =KCTC 42351^T^ =CPCC 100614^T^) (also known as *Xanthomonadaceae* bacterium 3.5X) (1) was originally isolated from an oil field in Shandong Province (China) during a study on the characterization of halophilic bacteria that show optimal polycyclic aromatic hydrocarbon (PAH)-degrading activity in petroleum-contaminated soil. The strain was isolated using PAHs as the sole carbon source on an agar plate; however, it showed no ability to degrade PAHs in liquid medium. The phylogenetic position of the strain was determined to be within the family *Xanthomonadaceae* and designated a new genus and species (1). To illustrate its potential role in degrading PAHs as well as other hydrocarbons, the genome of this strain was sequenced.

Genomic DNA from the strain was extracted and precipitated following the cetyltrimethylammonium bromide (CTAB) protocol (2). The whole-genome sequence of strain 3.5X was performed using Illumina MiSeq technology (Major bio, China) after the construction of two sequencing libraries, a 400-bp paired-end library and a 3,000-bp mate-pair library. In total, 787.1 Mbp (paired-end) and 380.0 Mbp (mate-pair) filtered data, which correspond to an average coverage of 232.8× and 112.4×, respectively, were generated. The raw reads from the two libraries were assembled together using SOAPdenovo version 1.05 (3) and GapCloser (3), yielding 22 contigs with the largest length of 883,955 bp and *N*_50_ of 389,485 bp. These contigs were further arranged into 6 scaffolds, with the largest length of 3,072,697 bp. The total assembly length of the genome is 3,379,958 bp, and the G+C content is 63.8%.

Glimmer 3.0 (4) was used to predict open reading frames (ORFs). The potential function of these predicted ORFs was annotated by using BLASTp (5) against several databases including NCBI-NR, the KEGG database (6), and the GO database (7). The rRNA and tRNA gene predictions were separately conducted by RNAmmer (8) and the tRNAscan-SE program (9). The determined draft genome contains 3,010 ORFs, within which only 1,647 genes have predicted functions in the KEGG database. Three rRNA genes (16S, 23S, and 5S rRNA) and 46 tRNA genes were predicted.

From the metabolic pathway predicted by KEGG, several genes related to hydrocarbon degradation were predicted, e.g., genes encoding methyltransferase-like protein in the PAH degradation pathway and phenol 2-monooxygenase for toluene or resorcinol degradation. However, these genes just play peripheral roles in hydrocarbon degradation, while key genes, like aromatic dioxygenase and alkane mono-oxygenase, are not present in the genome. It seems that this strain cannot utilize hydrocarbons in oil as substrates directly. Conversely, gene annotation revealed the existence of 25 genes related to a bacterial secretion system and 13 genes associated with protein export, corresponding to the appearance of sticky colloids around the strain colony on the agar plate. These results indicate that this strain has the ability to secrete extracellular polymeric substances, which may facilitate hydrocarbon degradation by other microbes.

### Nucleotide sequence accession numbers.

This whole-genome shotgun project has been deposited at DDBJ/EMBL/GenBank under accession no. JROI00000000. The version described in this paper is version JROI00000000.1.
